# Computer-Aided Drug Design in Epigenetics

**DOI:** 10.3389/fchem.2018.00057

**Published:** 2018-03-12

**Authors:** Wenchao Lu, Rukang Zhang, Hao Jiang, Huimin Zhang, Cheng Luo

**Affiliations:** ^1^Drug Discovery and Design Center, CAS Key Laboratory of Receptor Research, State Key Laboratory of Drug Research, Shanghai Institute of Materia Medica, Chinese Academy of Sciences, Shanghai, China; ^2^Department of Pharmacy, University of Chinese Academy of Sciences, Beijing, China; ^3^School of Life Science and Technology, ShanghaiTech University, Shanghai, China

**Keywords:** drug discovery, epigenetics, small-molecule inhibitor, computer-aided drug design, virtual screening

## Abstract

Epigenetic dysfunction has been widely implicated in several diseases especially cancers thus highlights the therapeutic potential for chemical interventions in this field. With rapid development of computational methodologies and high-performance computational resources, computer-aided drug design has emerged as a promising strategy to speed up epigenetic drug discovery. Herein, we make a brief overview of major computational methods reported in the literature including druggability prediction, virtual screening, homology modeling, scaffold hopping, pharmacophore modeling, molecular dynamics simulations, quantum chemistry calculation, and 3D quantitative structure activity relationship that have been successfully applied in the design and discovery of epi-drugs and epi-probes. Finally, we discuss about major limitations of current virtual drug design strategies in epigenetics drug discovery and future directions in this field.

## Introduction

Covalent modifications on nucleosomes, the basic building blocks on chromatins, including methylation, acetylation, phosphorylation, and ubiquination specifically regulate downstream gene expression patterns in a context-dependent manner that form the fundamental molecular basis of epigenetics (Strahl and Allis, 2000; Berger, 2007). Dynamic regulation of epigenetic modification collections leads to different functional outcomes that plays a pivotal role in biological processes including genome reprogramming, gene transcription, DNA damage response and homeostatic regulation (Li, 2002; Vidanes et al., 2005; Kouzarides, 2007; Gut and Verdin, 2013). Epigenetic dysfunction is tightly related with the pathogenesis and progression of several diseases including malignant diseases especially cancers and chronic diseases such as immune-mediated diseases, neurodegenerative disorders and diabetes which underscoring the importance of these covalent modifications (Best and Carey, 2010; Dawson and Kouzarides, 2012; Tough et al., 2016; Hwang et al., 2017).

Proteins responsible to modulate epigenetic marks on nucleosomes could be roughly divided into three categories based on their relative function including writers (enzymes that deposit covalent modifications), erasers (enzymes that remove covalent modifications), and readers (proteins that recognize specific modifications and recruit chaperons). Encouraging success has been achieved in the development of epi-probes for dissecting epigenome in recent decades (Shortt et al., 2017). However, there is still formidable challenge for epigenetic drug discovery in both academia and industry due to complexity in epigenetics regulatory network and the limits in assays and drug development technologies. So far only seven epigenetic agents targeting two epigenetic enzymes (DNA methytransferases, histone deacetylases) have been approved for human use. The indications of approved epigenetic drugs are limited to malignant diseases such as myelodysplastic syndromes (MDS), acute myeloid leukemia (AML), chronic myelomonocytic leukemia (CML), peripheral T-cell lymphoma (PTCL), and cutaneous T-cell lymphoma (CTCL) while the applications of epigenetic drugs in chronic diseases treatment were less explored (Mann et al., 2007; Derissen et al., 2013; Laubach et al., 2015; Lee et al., 2015). Hence, there is urgent need to develop novel epigenetic drugs with multidisciplinary efforts and extensive collaborations that may accelerate the pace of drug discovery process.

**Figure 1:**
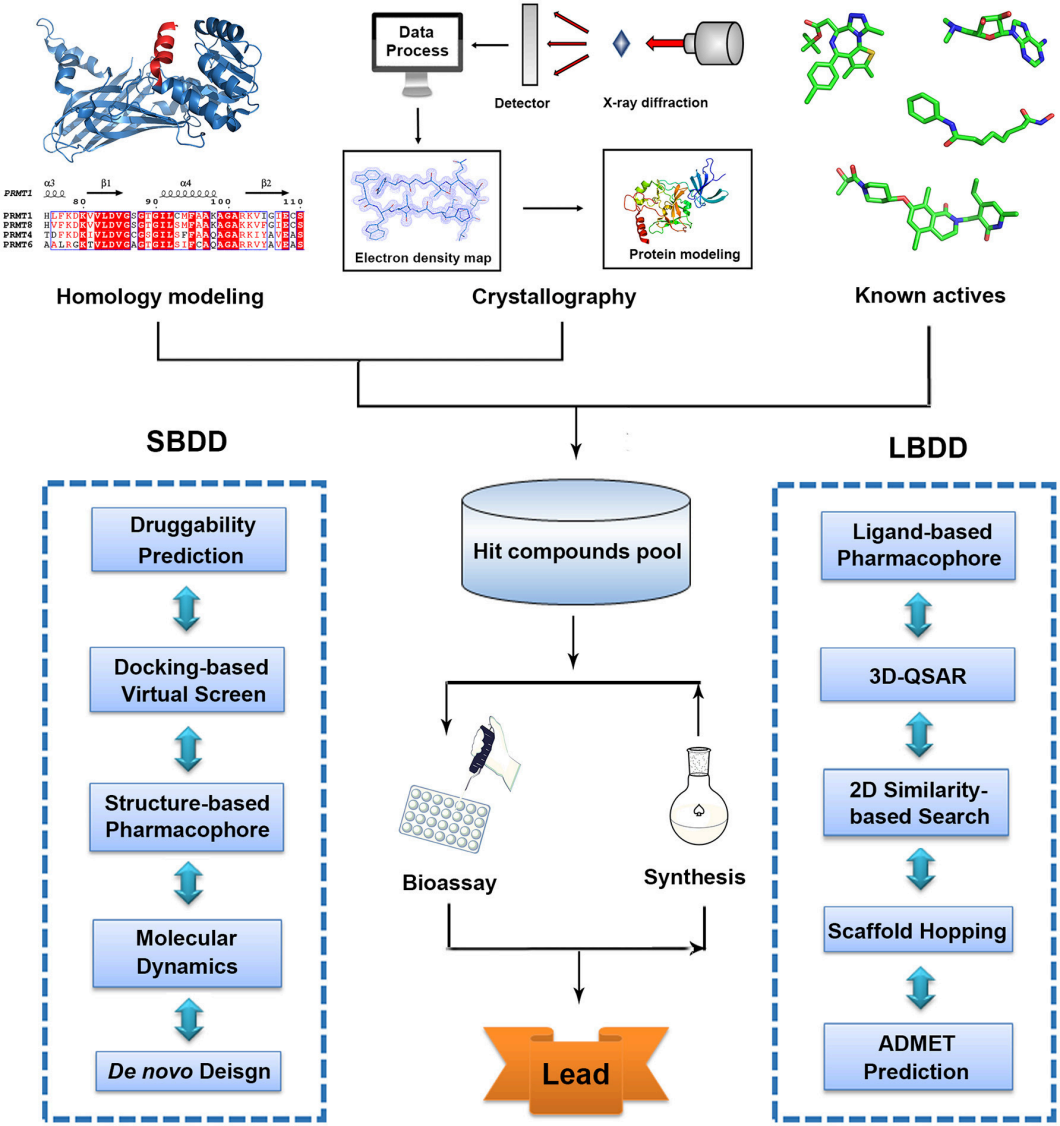
Traditional workflow of structure-based drug design (SBDD) and ligand-based drug design (LBDD).

With advanced development of computational methodologies, computer-aided drug design (CADD) has emerged as a burgeoning research filed (Zheng et al., 2013). Currently, many pharmaceuticals companies and research institutions all over the world have established their own CADD departments and continued efforts have been made toward the development and optimization of drug design methodologies and software (Kim et al., 2017). *In silico* druggability assessment helps researchers to identify more chemical-tractable targets and prioritize screening endeavor (Trosset and Vodovar, 2013). Based on rapid advancement of crystallography and successful applications of homology modeling, structure-based virtual screen (SBVS) has proven a useful method to quickly identify bioactive hits in early-stage discovery activities (Lounnas et al., 2013). Ligand-based drug design (LBDD) strategies like three dimensional quantitative structure activity relationship (3D-QSAR), 2D similarity-based searching, scaffold hopping and pharmacophore studies are also efficient approaches for hit enrichment and activity prediction based on available information of known inhibitors (Meena et al., 2011; Andrew et al., 2016; Yadav et al., 2017b). Moreover, quantum mechanical calculation and molecule dynamic (MD) simulation provide the in-depth understanding in protein catalytic mechanism that is quite useful mechanism-based drug design (Scheraga et al., 2007). *In silico* pharmacokinetic properties assessment allows the prediction of absorption, distribution, metabolism, elimination, and toxicity (ADMET) of drug candidates that is an important cheminformatics tool in drug design (Gaur et al., 2015; Yadav et al., 2016). Collectively, combined with the gained availability of diverse compound databases, these cost effective structure-based or ligand-based strategies significantly increase the efficiency in drug discovery and provide new horizons and promising avenues to conquer life-threatening diseases (Figure 1, Table 1).

**Table 1:** The online public and commercial databases and compound collections used for virtual screening in epigenetics.

**Database**	**Size**	**Website**	**Applications**
ZINC	Over 35 million	http://zinc.docking.org	GCN5; KDM4A/C; HDAC1; BRD4/T; SIRT2-3
SPECS	~320,000	http://www.specs.net	DOT1L; DNMT1; SET7; DNMT3A; PRMT1; G9a; FTO; PRMT5; EZH2; HDAC8; LSD1; BRD4; Menin-MLL1
NCI	~260,000	https://cactus.nci.nih.gov/	DNMT1; Class I/IIa HDACs; KDM4A/B; BRD2
Maybridge	~56,000	http://www.maybridge.com	LSD1; HDAC2; HDAC8
ChemBridge	~1.1 million	http://www.chembridge.com	Type I PRMTs; PRMT5; p300/CBP; BRD4; Spindlin1; SIRT3
CoCoCo	~7 million	http://cococo.isof.cnr.it	SMYD3
Enamine	~2.4 million	http://www.enamine.net	LSD1; PRMT5
ChEMBL	~1.7 million	http://www.ebi.ac.uk/chembldb/index.php	GCN5; BRD4
ChemDiv	~1.5 million	http://www.chemdiv.com	PRMT5; BRD4; Spindlin1
Dictionary of Natural Products	~40,000	http://dnp.chemnetbase.com	BRD4
ASINEX	~87,000	http://www.asinex.com/libraries_synergy.html/	HDAC1
InterBioScreen	~550,000	https://www.ibscreen.com	HDAC1
eMolecules	Over 8 million	http://www.emolecules.com	BRD4; WDR5-MLL1
Life chemicals	~1.35 million	http://www.lifechemicals.com	BRD4
DrugBank	~10,000	http://www.drugbank.ca	SIRT3
WDI	~80,000	http://www.daylight.com/products/wdi.html	HDAC1; HDAC6

*Data accessed in December 28, 2017*.

Although these leading computational strategies have been successfully applied in traditional drug discovery pipeline, there are relatively few reports focusing on its contribution in epigenetic landscape (Li et al., 2015). In this review, we mainly focus on recent progress on the applications of these strategies and highlight representative studies and major contributions of computational approaches in this field. Other successful drug discovery studies using wet lab approaches are beyond the field of this review and not covered here that may also be interesting aspects in epigenetic-related studies.

## Writer

**Figure 2:**
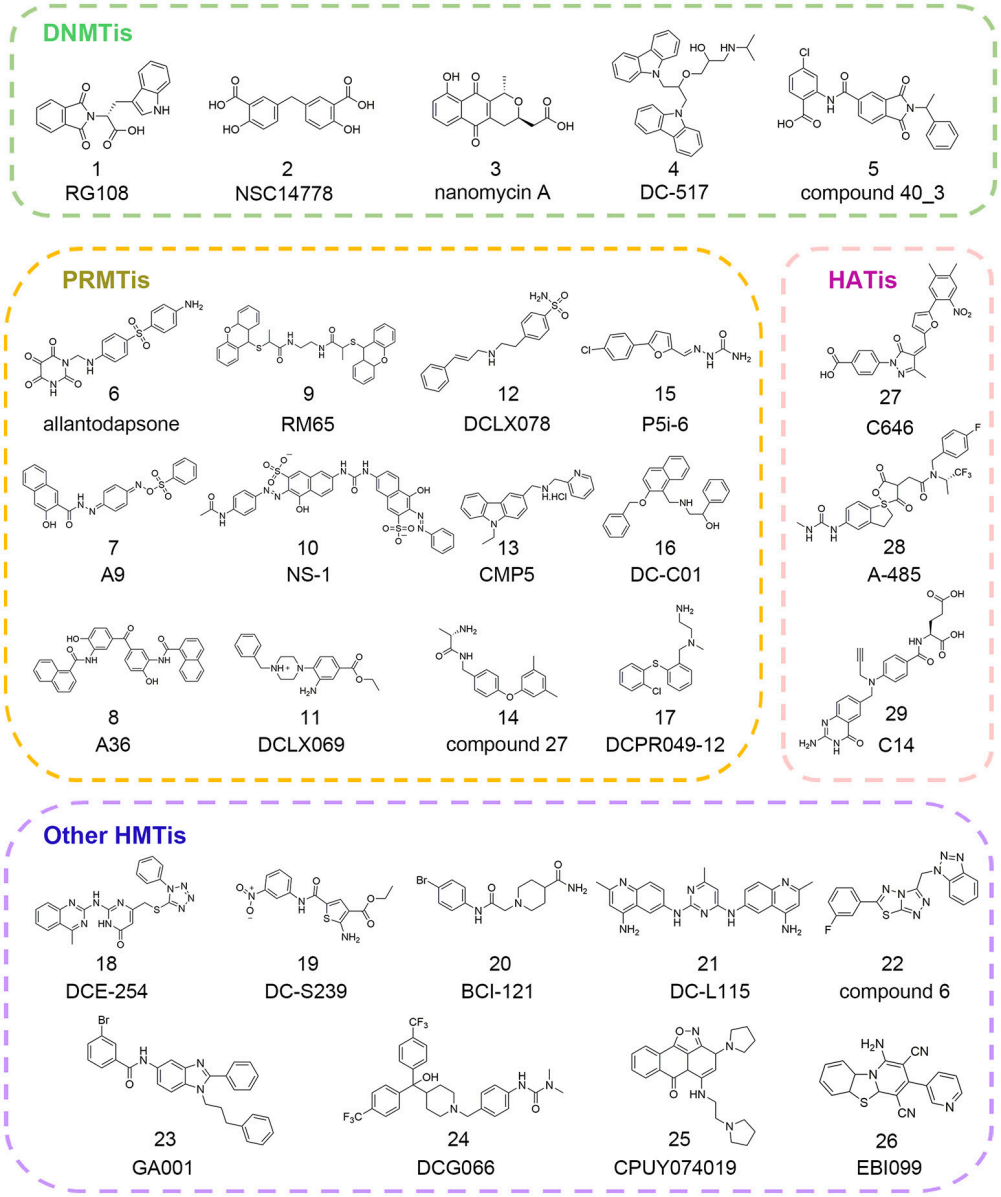
Chemical structures of epigenetic writer inhibitors mentioned in this review.

Epigenetic writers are the enzymes responsible for transferring methyl groups or acetyl groups to DNA, histone or other non-histone substrates from cofactors *S*-adenosyl-_L_-methionine (SAM) or acetyl coenzyme A (Ac-CoA). Based on their distinct functions, writers are usually divided into three categories, namely DNA methyltransferases (DNMTs), protein lysine/arginine methyltransferases (PKMTs/PRMTs) and histone acetyltransferases (HATs). These enzymes alter chromatin organization and contribute to downstream gene expression regulation through site-specific modification that are involved in the multiple function pathways (Gelato and Fischle, 2008). To elucidate their roles in physiological or pathological states, there has been increasing interest in the discovery of writer inhibitors through *in silico* approaches and many successful stories have been reported in the literature (Figure 2). In this section, we will present an overview of the current applications of computational methods used in hit identification targeting epigenetic writers.

### DNA methyltransferases

DNA methyltransferases catalyze DNA methylation by depositing a methyl group on the 5-position of the cytosine (Robertson, 2001). In mammalian cells, there are five members identified so far: DNMT1, DNMT2, DNMT3A, DNMT3B, and DNMT3L. Among them, DNMT1 is characterized as the maintenance methyltransferase that shows preference for hemimethylated DNA substrates while DNMT3A and DNMT3B belong to *de novo* methyltransferases subfamily that function in complex form and catalyze the methylation of unmethylated DNA (Okano et al., 1999; Goll and Bestor, 2005). In DNMTs, the founding member DNMT1 is the best studied. DNMT1 introduces a new methyl group into newly synthesized DNA strand in the context of CpG dinucleotide that maintains methylation patterns of template strand during DNA replication (Bestor, 2000; Auclair and Weber, 2012). Aberrant promoter DNA hypermethylation leads to silencing of tumor suppressor genes, which has been frequently observed in various carcinomas (Feinberg et al., 2006; Zhang and Xu, 2017). Therefore, DNMTs have become one of the most promising targets for cancer therapy and many computational approaches have evolved to fuel the development of epi-probes and epi-drugs targeting DNMTs (Medina-Franco et al., 2015).

#### Homology modeling-driven studies

Homology modeling is a quite effective strategy especially when interested protein crystal structures are not available and it functions as the most valuable research tool to fill the sequence-structure gap for structure-based drug design (Dwivedi et al., 2015). The accuracy of homology models mainly depends on the sequence identity or similarity between the template protein and the protein to be modeled (Chothia and Lesk, 1986). As commonly accepted, homology models based on more than 50% of sequence identity with proteins whose structures have been experimentally acquired are usually very accurate and can be used for drug discovery purposes (Hillisch et al., 2004).

Since there was no DNMT1 crystal structure ever released until 2008, drug development against this therapeutic target progressed slowly (Syeda et al., 2011). To circumvent this, Siedlecki et al. built a homology model of human DNMT1 catalytic domain based on available structural information of M.HhaI, M.HaeIII, and DNMT2 in the MODELLER module of INSIGHT2000 (Siedlecki et al., 2003). In a follow-up study, based on this established homology model, Siedlecki and co-workers performed docking-based virtual screening of a diversity set containing 1,990 compounds from the National Cancer Institute (NCI) that represented more than 140,000 compounds using DOCK version 5.1.0. The screen resulted in the discovery of RG108 (compound 1 in Figure 2) that came out on top in biochemical assays (Brueckner et al., 2005; Siedlecki et al., 2006).

Similarly, Kuck et al. carried out virtual screening (VS) of a larger data set including more than 65,000 lead-like compounds based on the aforementioned DNMT1 homology model. Top-ranked compounds were re-scored by GLIDE, GOLD, and AUTODOCK followed by experimental tests. Among them, NSC14778 (compound 2 in Figure 2) presented inhibitory activities against DNMT1 and DNMT3B with the IC_50_ value of 92 and 17 μM, respectively while nanaomycin A (compound 3 in Figure 2) selectively inhibited DNMT3B with the IC_50_ value of 500 nM. To explain the selective inhibitory activities of nanaomycin A, the authors established homology model of DNMT3B catalytic domain based on DNMT3A crystallographic structure, which provided structural basis for mechanism interpretation (Kuck et al., 2010a,b).

In order to further disclose the mechanism of action (MOA) of nanaomycin A, Caulfield and co-workers performed >100 ns molecular dynamic simulation using the CHARMM27 force field in NAMD version 2.62. The previously established DNMT3B homology model bound to nanaomycin A was used with either presence or absence of cofactor SAM in the simulation. The results suggested that nanaomycin A and SAM could bind to DNMT3B in a cooperative manner. Besides, nanaomycin A could form long-lasting interactions with key residues that involved in the methylation process which further validated the hypothesis supported by previous docking simulation (Caulfield and Medina-Franco, 2011).

#### High throughput virtual screening

In 2014, through docking-based virtual screening based on the complex structure of mouse DNMT1 bound to *S*-adenosyl-_L_-homocysteine (SAH) (PDB ID: 4DA4), Chen et al. reported a novel non-nucleoside DNMT1 inhibitor DC_05 that showed significant selectivity toward other protein methyltransferases (Chen S. et al., 2014). Further medicinal chemistry optimization led to the discovery of more potent compound DC_517 (compound 4 in Figure 2) with the IC_50_ value of 1.7 μM. The putative binding models were generated based on molecular docking studies, which gave detailed interpretation of the structure-activity relationship (SAR).

In 2017, in order to identify novel DNMT3A inhibitors, Shao et al. conducted a multi-step docking-based virtual screening in combination with pharmacophore mapping. Through initial screening and follow-up similarity-based analog searching, the authors discovered novel DNMT3A inhibitor compound 40_3 (compound 5 in Figure 2) with the IC_50_ value of 41 μM, which may serve as the starting point to develop more potent DNMT3A inhibitors (Shao et al., 2017).

#### Quantum mechanical calculation

In 2012, based on the *ab initio* methods, Alcaro and co-workers developed the force field parameters implemented in the MacroModel package for the treatment of charge distribution and overall charge assignment of nucleic acids that undergo methylation. It gives essential insights related to the correct charge treatment and force field parameterization, which is an important issue in molecular modeling of epigenetic phenomena and shed light for the nucleic acids-related epigenetic functional study and in the development of DNA intercalating, subtype-selective DNMTs inhibitors (Alcaro et al., 2002).

#### Histone methyltransferases

Histone methylation is one of the most important post-translational modifications on histones and results in either activation or repression depending on specific sites. The methylation marks could recruit different methyl-binding proteins and mediate downstream signaling pathways, which could be basically regulated by dynamic interplay between histone methyltransferases (HMTs) and demethyltransferases (HDMTs) (Martin and Zhang, 2005). Histone methyltransferases can be mainly divided into two categories based on their relative substrates: protein lysine methyltransferases and protein arginine N-methyltransferases (Li et al., 2012). Among them, PKMTs consist of SET domain-containing PKMTs (SUV, SET1, SET2, EZ, and RIZ) and non-SET domain-containing PKMT (DOT1L) (Kouzarides, 2002). As for PRMT family, it could be further classified into three subcategories: type I PRMTs responsible for arginine monomethylation and asymmetric dimethylation (PRMT1, 2, 4, 6, 8), type II PRMTs (PRMT5, 9) for arginine monomethylation and symmetrical dimethylation and type III PRMT (PRMT7) only with arginine monomethylation activity (Wolf, 2009). Emerging evidence demonstrated that deregulated alternations of methylation patterns were implicated in the pathogenesis of various cancers and other malignant diseases (Spannhoff et al., 2009; Jones et al., 2016). Consequently, continued efforts have been devoted to drug design for HMTs, which open up vast ranges of prospects for diseases treatment (Table 2).

**Table 2:** The HMTs inhibitors derived based on virtual screening or high throughput screening.

**Targets**	**Virtual screen**			**High throughput screen**		
	**I^a^**	**II^b^**	**III^c^**	**I**	**II**	**III**
PRMT1	★			★		
PRMT3	★			★		
PRMT4	★			★		
PRMT5	★			★		
PRMT6	★			★		
DOT1L		★		★		
SUV420H1	–	–	–	★		
SUV420H2	–	–	–	★		
SMYD2	–	–	–	★		
SMYD3			★	★		
GLP	–	–	–	★		
G9a		★		★		
NSD2			★	–	–	–
SETD2	–	–	–	★		
SETD7		★		★		
SETD8		★		★		
EZH1	–	–	–	★		
EZH2			★	★		

a *The IC_50_ values smaller than 1 μM*.

b *The IC_50_ values at the range of 1–10 μM*.

c *The IC_50_ values at the range of 10–100 μM*.

*The stars here denote that the IC_50_ value of the most potent compound against this target is in the corresponding range*.

#### Pharmacophore-based drug discovery

With the increasing knowledge of known active molecules available in databases, pharmacophore modeling methods are receiving more attention in the era of rational drug design that could quickly extract the key steric and electronic features for ligand-receptor interactions (Guner et al., 2004; Yadav et al., 2010, 2012). In 2007, Spannhoff et al. presented first target-based virtual screening with NCI diversity set to discover novel PRMT1 inhibitors. The GRID-based pharmacophore model, the methodology originally introduced by Ortuso et al. in 2006, was applied as post-docking filter to analyze all preliminary docking solutions (Ortuso et al., 2006). The study resulted in the identification of allantodapsone (compound 6 in Figure 2) with the IC_50_ value of 1.7 μM (Spannhoff et al., 2007a).

In the follow-up study, Heinke et al. expanded their work to a larger compound collection, the ChemBridge database containing 328,000 molecules (Heinke et al., 2009). Based on previously reported binding modes of allantodapsone, the pharmacophore models were generated in LigandScout with one HBD, one hydrogen bond acceptor (HBA), two hydrophobic/aromatic features, one included volume and 23 excluded volumes leading to the identification of nine compounds with PRMT1 inhibitory activity below 35 μM.

Ligand-based pharmacophore modeling is also a powerful tool in drug discovery campaigns. In 2012, Wang et al. constructed four rational pharmacophore models with HBA, HBD, and ring/aromatic (RA) as key chemical features based on 17 reported active molecules in Discovery Studio version 2.1. Then established models were used as the query to search theoretical-soluble small molecule library. Through cluster analysis in combination with biological assays, A9 and A36 (compounds 7–8 in Figure 2) were identified as PRMT1 inhibitors with the IC_50_ values of 41.7 and 12.0 μM, respectively (Wang et al., 2012). Kinetic analysis demonstrated that A9 was a peptide-competitive PRMT1 inhibitor whereas A36 was the non-competitive PRMT1 inhibitor that could be used as the parent compounds for further chemistry optimization.

The pharmacophore modeling has also been widely applied in hit identification targeting other HMTs. In 2016, aiming to identify novel EZH2 inhibitors, Wu et al. conducted ligand-based pharmacophore modeling based on validated EZH2 inhibitors (Wu et al., 2016). The reliability of constructed models was evaluated by enrichment capacity analysis using molecules in test sets. Based on the established models, they identified novel EZH2 inhibitors DCE_254 (compound 18 in Figure 2) with the IC_50_ value of 11 μM.

In 2015, through integrated structure-based pharmacophore-modeling and molecular docking, Meng et al. discovered a SET7 inhibitor, namely DC_S100 with the IC_50_ value of 30.0 μM (Meng et al., 2015). Docking-based SAR analysis followed by structure optimization led to the identification of DC-S239 (compound 19 in Figure 2), with the IC_50_ value of 4.6 μM. In addition, DC-S239 could dose-dependently inhibit the proliferation of MCF7, HL60, and MV4-11 with the IC_50_ values at micromolar range supporting its potential use in cellular context.

#### Molecular dynamics simulation

Molecular dynamics simulation is a useful theoretical technique to investigate the conformations and dynamic behaviors of biomolecules in long-time scale that provides atomic-level insight into the regulatory mechanism (Lindorff-Larsen et al., 2011; Okumura et al., 2018). To characterize the elusive roles of the N-terminal region and dimerization arms for PRMT1 activity, Zhou et al. performed MD simulations using GROMACS 4.3 package based on hPRMT1 homology model in monomer and dimer states (Zhou et al., 2015b). The simulations captured the dynamic correlations between the N-terminal region and dimerization arms. Moreover, the normalized covariance analysis and principal component analysis (PCA) were applied to analyze the energy landscape of different conformations at reduced dimensions. Through network topology analysis, a long-distance communication pathway was theoretically proposed which was further validated by biochemical mutational experiments. The simulations disclosed the underlying molecule mechanism of allosteric communication between the two regions and provided the rationale for mechanism-based PRMT subtype-selective inhibitors.

Molecule dynamic simulations could be not only wildly applied in protein dynamic regulation studies but also in MOA studies for small molecule inhibitors. In order to uncover the molecular basis of diamidine inhibitors for selective PRMT1 inhibition, Yan et al. conducted extensive MD simulations and molecular mechanics/Poisson–Boltzmann solvent-accessible surface area (MM/PBSA) calculation to analyze the interaction patterns in the binding cavity for the docking complex which provided the avenue to design more potent and specific inhibitors (Yan et al., 2014; Zhang et al., 2017a,b). A similar MD study was reported by Yang and co-workers to propose binding poses of identified PRMT1 inhibitors and circumvent the limitations introduced by inaccuracy of molecule docking methods (Yang et al., 2017).

#### High throughput virtual screening

The big explosion of available structural information of HMTs has greatly facilitated the application of docking-based virtual screening (DBVS). In 2007, via virtual screening and 2D similarity-based analog searching, Spannhoff et al. identified RM65 (compound 9 in Figure 2) as the PRMT1 inhibitor with the IC_50_ value of 55.4 μM (Spannhoff et al., 2007b). In 2010, a similar study was reported by Feng and co-workers in structure-based virtual screening with 400,000 compounds. In this study, NS-1 (compound 10 in Figure 2) was identified as the PRMT1 inhibitor with an IC_50_ value of 12.7 μM which directly targeted the peptide substrates instead of enzymes (Feng et al., 2010). In 2014, Xie and co-workers used the combinatorial dockings methods including GLIDE and DOCK for *in silico* screen. The authors identified DCLX069 and DCLX078 (compounds 11–12 in Figure 2) with the IC_50_ values of 17.9 and 26.2 μM, respectively in biochemical assays (Xie et al., 2014).

A number of attempts have also been made to identify other PRMTis besides PRMT1 inhibitors. In 2015, Alinari et al. used comparative modeling and structure-based virtual screening with ChemBridge CNS-Set library of 10,000 small molecule compounds leading to the identification of first-in-class PRMT5 inhibitor CMP5 (compound 13 in Figure 2). In cellular context, CMP5 could selectively inhibit the proliferation and transformation of EBV-driven B-lymphocyte (Alinari et al., 2015). In 2016, Ferreira et al. started their work based on the two basic amine tails that mimicked the side chain of substrate arginine and established PRMT-focused virtual library. Through initial biochemical screening and structure-based optimization, the authors identified compound 27 (compound 14 in Figure 2) as the selective CARM1 inhibitor with an IC_50_ value of 0.05 μM and ligand efficiency of 0.43 (Ferreira de Freitas et al., 2016). In 2017, Ji et al. carried out molecular docking studies with semi-flexible docking methods in GOLD and identified selective PRMT5 inhibitor P5i-6 (compound 15 in Figure 2) with an IC_50_ of 0.57 μM (Ji et al., 2017). Similarly, Ye et al. identified PRMT5 inhibitor named DC_C01 (compound 16 in Figure 2) with the IC_50_ value of 2.8 μM via docking-based virtual screening and structure modification (Ye et al., 2017). Concurrent with the two studies described above, through hierarchical docking strategies and chemistry optimization, Chen et al. identified DCPR049_12 (compound 17 in Figure 2) with promising inhibitory activity for type I PRMT with the IC_50_ value at nanomolar range (Wang et al., 2017).

Besides PRMTs, docking-based virtual screening strategy was also applied in the epi-probe design for other HMTs. In 2015, in an attempt to search for SMYD3 inhibitors, Peserico et al. performed high-throughput virtual screening of the CoCoCo database containing nearly 260,000 molecules in GLIDE version 5.7 (Peserico et al., 2015). The study led to the identification of BCI-121 (compound 20 in Figure 2) as the best candidate for SMYD3 inhibition that could reduce global H3K4me2/3 and H4K5me levels in colorectal cancer. Similarly, Chen et al. identified the DOT1L inhibitor DC_L115 (compound 21 in Figure 2) with an IC_50_ value of 1.5 μM via structure-based virtual screening of approximately 200,000 molecules in SPECS database (Chen S. et al., 2016).

Very recently, Wang and co-workers developed a target-specific scoring function based on epsilon support vector regression (ε-SVR) named the SAM-score for SAM-dependent methyltransferases. Based on the built regression model, the authors identified compound 6 (compound 22 in Figure 2) as the DOT1L inhibitor with an IC_50_ of 8.3 μM (Wang et al., 2017). There are also some successful studies reported elsewhere for the discovery of other HMT inhibitors (compounds 23–26 in Figure 2) for G9a and SETD8 based on *in silico* approaches (Chen W. L. et al., 2016; Kondengaden et al., 2016; Milite et al., 2016).

### Histone acetyltransferases

Histone acetyltransferases (HATs) transfer acetyl groups onto N-terminal tails of core histone and consequently give rise to DNA relaxation, which is closely related to gene activation (Brown et al., 2000). HATs can be divided into four categories on the basis of their sequence similarities, including the GNAT family (GCN5 and PCAF), the MYST family (MOZ/MORF, YBF2/SAS3, SAS2, and TIP60), p300/CBP and RTT109 (Dancy and Cole, 2015). Recently, emerging evidence implicated that deregulation of HATs was closely correlated with tumorigenesis, neurological disorders and inflammatory diseases (Yang, 2004; Rajendrasozhan et al., 2009; Sheikh, 2014). Several HAT inhibitors have been reported, such as bi-substrate inhibitors, natural products, and small molecules. However, there is still a large gap between activities *in vitro* and their potential applications as therapeutic agents *in vivo* due to the lack of potency and selectivity for the current inhibitors which is a long-standing challenge in the field.

In 2010, Bowers et al. conducted structure-based, *in silico* screening approach with a screening set of *ca*. 500,000 commercially available compounds to identify the p300 inhibitor (Bowers et al., 2010). The compounds were scored and ranked based on ICM (Internal Coordinate Mechanics) score in the ICM-VLS software version 3.5. Then top 194 compounds were cherry-picked by visual inspection and purchased from ChemBridge for biochemical analysis. Among them, C646 (compound 27 in Figure 2) was identified with *K*i value of 400 nM. Further *in vitro* assay demonstrated that C646 was cofactor-competitive and selective p300 inhibitor. The detailed interaction patterns were confirmed by site-directed mutagenesis in accordance with the predicted computational model.

Very recently, Lasko and co-authors performed similar docking-based *in silico* screening with nearly 800,000 compounds and 1,300 available compounds were test in radioactive p300 acetylation assays (Lasko et al., 2017). Among them, hydantoin and a conjugated thiazolidinedione were identified with the IC_50_ values of 5.1 and 11.5 μM, respectively. More efforts were devoted to the optimization on hydantoin scaffold yielding A-485 (compound 28 in Figure 2) with an IC_50_ value of 60 nM. A-485 was a first-in-class highly potent, selective p300/CBP catalytic inhibitor and displayed significant selectivity against other HATs members. Besides, it inhibited proliferation across a broad range of cancer cell lines with specificity for hematological and prostate cell lineages and retarded tumor growth in xenograft models, which underscored the therapeutic potential targeting p300/CBP. Another small molecule discovered by virtual screening from ChEMBL bioassay database was C14 (compound 29 in Figure 2) with an IC_50_ value of 225 nM on PfGCN5 in parasite growth assay (Kumar et al., 2017). C14 displayed promising antimalarial activity and showed no effect on mammalian fibroblast cells supporting its safe use for further applications.

## Eraser

**Figure 3:**
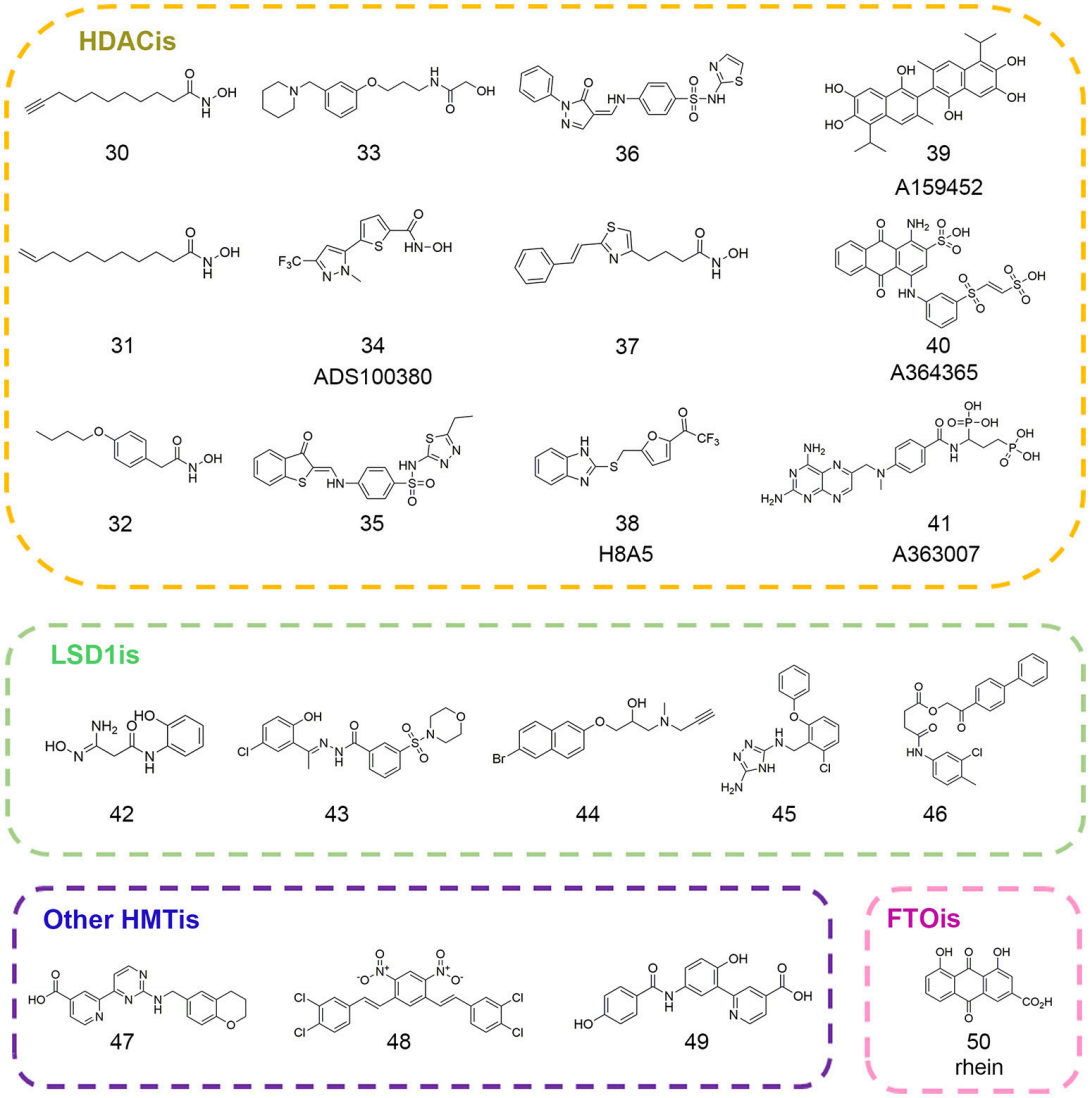
Chemical structures of epigenetic eraser inhibitors mentioned in this review.

Erasers are key modifying enzymes in charge of the removal of epigenetic marks that participate in dynamic regulation on gene expression patterns (Mosammaparast and Shi, 2010). Based on their different substrates and their relative functions, erasers could be divided into different families such as histone deacetylases (HDACs), RNA demethyltransferases, histone demethylases (HDMs), histone deubiquitinases, and so on (Arrowsmith et al., 2012). Among them, HDACs are the most studied targets for pharmacological interventions. So far, five epi-drugs targeting HDACs have been approved for clinical use and other HDAC inhibitors like entinostat and CUDC-907 have entered into clinical trials for advanced cancer treatment (Falkenberg and Johnstone, 2014; Li and Seto, 2016). In the following section, we will focus on representative computational work in drug discovery and related mechanism studies that expert in this field (Figure 3).

### Histone deacetylases

In mammalian cells, HDACs consist of 18 isoforms and are broadly classified into four categories based on their distinct structural features and subcellular localization: Class I (HDAC1, 2, 3, and 8), Class II (Class IIa HDAC4, 5, 7, 9, and Class IIb HDAC6, 10), Class III (NAD-dependent Sirtuins; SIRT1-7) and Class IV (HDAC11) (Gregoretti et al., 2004; Li and Seto, 2016). HDACs catalyze the deacetylation of histone as well as non-histone substrates and are implicated in fundamental physiological processes including gene transcription, cell cycle regulation, DNA damage response, and metabolism homeostasis (Bode and Dong, 2004; Minucci and Pelicci, 2006). There is a growing body of evidence that deregulation of HDACs activity is strongly correlated with the pathogenesis of several diseases including hematological malignancies and solid tumors that implicates the significance of target intervention (Minucci et al., 2001; Zhu et al., 2004; Buurman et al., 2012). Significant progress has been made in the development of HDAC inhibitors (HDACis) over the recent decades based on *in silico* approaches (Yanuar et al., 2016). The following chapters will focus on some representative studies using computational methods in this field, some of which are described below.

#### Quantum mechanical calculation

Hydroxamic acid moiety presented in most common HDACis is usually recognized as problematic fragment with poor pharmacokinetic profile. To rationally design non-hydroxamic acid HDACis with more favorable physico-chemical properties, Wang et al. performed density functional theory (DFT) calculations to investigate binding modes and related binding free energy of potential zinc binding groups (ZBGs) (Wang et al., 2007). In model active site, only the side chains of zinc-coordinated residues were kept for calculation including two formats and one imidazole that represented as the functional groups of zinc-coordinated histidine and two aspartic acid residues. The calculation results proposed alternatives with novel structural features that favored zinc binding including 3-hydroxy pyrones or β-amino ketones, which may be further utilized for medicinal chemistry optimization on current HDACis.

Apart from the applications in novel hit discovery, quantum mechanical calculation could also enable precise and solid interpretation into mechanism studies that facilitates the drug design of novel and specific HDACis. Finin et al. proposed that H143-D183 catalytic dyad was indispensable for HDAC8 enzymatic activity by abstracting proton from the bridged water molecule while Zhang et al. underscored the role of H142-D176 dyad in proton-shuttle process (Finnin et al., 1999; Wu et al., 2010). In addition, the controversial function of potassium ion near the active pocket present in HDAC crystal structures is also under debate (Gantt et al., 2010; Werbeck et al., 2014). Based on QM/MM simulations including the complete catalytic residues in the quantum region, Chen et al. explained disagreement for those observations and uncovered the unique catalytic mechanism of HDAC8. The results disclosed the inhibitory role of the potassium ion at the active site and uncovered the significance of the p*K*_a_ values of zinc-coordinated moiety in HDACis that would be of great value in developing potent and subtype-selective mechanism-based HDACis (Chen K. et al., 2014).

#### Quantitative structure-activity relationship analysis

QSAR analysis is a well-established ligand-based computational methodology to describe the quantitative relationship between compound biological activity and its physicochemical properties or structural features, which is the milestone progress in the era of rational drug design (Gupta, 2007; Yadav et al., 2013, 2017a). Since therapeutic value of HDACis has been addressed over recent years and many potent HDACis have been identified so far, comprehensive QSAR studies were conducted using different kinds of data sets to facilitate drug design and discovery against this drug-actionable target. In 2004, Wang et al. developed QSAR models based on hydroxamic acid-based HDACis and found statistically significant relationship between charge distribution, hydrophobicity, geometrical shape of compounds and its relative anti-proliferative activities for PC-3 cell lines (Wang et al., 2004). Since then, the number of QSAR modeling studies increased at a dramatic rate (Xie et al., 2004; Guo et al., 2005; Juvale et al., 2006; Chen et al., 2008; Kozikowski et al., 2008; Ragno et al., 2008).

The first QSAR studies used for virtual screening was reported by Tang and co-workers (Tang et al., 2009). Based on validated QSAR models, the authors screened the in-house library with *ca*. 9.5 million compounds and identified four novel scaffolds that favored HDACs inhibition (compounds 30–33 in Figure 3). In 2012, Xiang and colleagues developed pharmacophore and 3D-QSAR models on a series of (benz)imidazole inhibitors (Xiang et al., 2012). The results led to the discovery of 27 inhibitors with putative HDAC2 inhibitory activity. Later on, several groups employed QSAR modeling workflow for HDACis activity and selectivity prediction (Silvestri et al., 2012; Zhao et al., 2013b). In 2014, Kandakatla et al. conducted ligand based 3D-QSAR pharmacophore modeling and identified eight hit compounds from Maybridge and NCI databases as potential HDAC2 inhibitors (Kandakatla and Ramakrishnan, 2014). In the same year, based on 79 previously published substrate-based SIRT1 inhibitors, Kokkonen and coworkers performed CoMFA studies that was successfully applied in the bioactivity prediction of 13 newly synthesized compounds (Kokkonen et al., 2014). Similarly, Cao et al. developed QSAR models using support vector classification and regression with scrupulous examination based on published HDAC8 inhibitors that was applied in next-round drug screening (Cao et al., 2016).

#### High throughput virtual screening

In 2007, Price and colleagues initiated virtual screening with HDAC-focused library containing 644 hydroxamic acids (Price et al., 2007). The study resulted in the identification of ADS100380 (compound 34 in Figure 3) with an IC_50_ value of 0.75 μM followed by iterative optimization. Similarly, another successful application of structure-based virtual screen for the discovery of HDACis was carried out by Park et al. based on HDAC1 homology model (Park et al., 2010). The newly identified inhibitors (compounds 35–36 in Figure 3) presented novel chemotypes that had not yet been reported before with IC_50_ values at micromolar range. In 2016, Yoo et al. rationally designed selective HDAC6 inhibitors with the IC_50_ value of 0.199 μM (compound 37 in Figure 3) inspired by preliminary virtual screening efforts with LeadQuest chemical database containing 80,600 entries (Yoo et al., 2016). Very recently, Hu designed a versatile VS pipeline with better screening power for the rapid discovery of selective HDAC3 inhibitors (Hu et al., 2017). Many efforts have also been devoted to the discovery of Sirtuins inhibitors, the NAD-dependent class III histone deacetylases that was reported elsewhere (Salo et al., 2013; Kokkonen et al., 2015; Padmanabhan et al., 2016).

As commonly accepted, each computational approach may not perform optimally when applied alone due to complexity of epigenetic network and this highlighted the importance of various combined *in silico* approaches in epigenetic drug discovery. Hou et al. developed ZBG-based pharmacophore model with enhanced sensitivity for virtual screening leading to the identification of selective HDAC8 inhibitor H8-A5 (compound 38 in Figure 3) with the IC_50_ value of 1.8 μM. Then molecular docking followed by 50 ns MD simulation was performed to give detailed insight of the MOA of identified hits (Hou et al., 2015). In 2017, Ganai and co-workers employed top-down combinatorial strategy of molecule docking and molecular mechanics generalized born surface area (MM-GBSA), MD simulation and trajectory clustering, energetically-optimized pharmacophore. The authors identified distinct hot spots in highly homologous HDAC1 and HDAC2 that shed light on the development of specific HDAC2 inhibitors against neurological diseases (Ganai et al., 2017). Hsu and co-workers employed VS approach against classified NCI database leading to the identification of class IIa-selective HDACis (compounds 39–41 in Figure 3). Homology modeling was performed to generate HDAC5 and HDAC9 3D structures that provide atomic-resolution insight into the selectivity of these inhibitors (Hsu et al., 2017).

### RNA demethyltransferases

RNA methylation is one of most important chemical marks in epigenetic landscape among which *N*^6^-methyladenosine (m6A) is the most abundant and conserved modification in eukaryotes (Desrosiers et al., 1974). Reversible *N*^6^-methyladenosine could be dynamically regulated by related writers and erasers involved in gene expression, RNA splicing, transport, and stability (Fu et al., 2014). Fat mass and obesity-associated (FTO) enzyme is one of the RNA demethylases and depends on Fe (II) and α-KG cofactors for its oxidative demethylation activity (Jia et al., 2011). Genetic variations of *FTO* are functionally associated with human obesity and metabolic disorders (Frayling et al., 2007). Recent studies demonstrate that FTO is highly expressed in MLL-rearranged AML and plays pivotal role in leukemogenesis (Li et al., 2017). Collectively, these studies hold promise for drug design and development targeting FTO for therapeutic translation.

In order to gain detailed insight into molecular mechanism for its catalytic specificity, the complex crystal structure of FTO and 3-meT substrate was resolved, which laid foundations for structure-based drug design (Yadav et al., 2010). Chen et al. employed virtual screening strategy in an effort to identify inhibitors targeting FTO active site. After initial screening against the drug-like SPECS database in Dock version 4.0, the primary results were evaluated in Sybyl and revisited by AutoDock version 4.0. Then top 300 compounds were selected for cluster analysis to ensure scaffold diversity. Finally, 114 compounds were picked out for biochemical validation leading to the identification of natural product rhein (compound 50 in Figure 3) as the competitive FTO inhibitor. Further decomposed binding energy prediction highlighted the electrostatic interactions between R316 and rhein, which was validated by follow-up biophysical studies (Chen et al., 2012; Aik et al., 2013). Later on, more efforts have been devoted to the drug design and discovery of selective FTO inhibitors (Huang et al., 2015; Toh et al., 2015). These identified structurally different inhibitor collections may serve as the parent templates applied in ligand-based drug design approaches. The small molecule sets could be used to establish focused and biased libraries that may be useful for rational drug design against other RNA demethylases.

### Histone demethyltransferases

Histone demethylation remained ambiguous until the hallmark discovery of first lysine specific demethylase LSD1 in 2004 (Shi et al., 2004). These demethyltransferases catalyze lysine/arginine demethylation and function as transcription corepressor that is tightly associated with dynamic regulation of methylation patterns shaping the epigenome (Dimitrova et al., 2015). Since then, more histone demethylases have been identified and their biological relevance has been disclosed (Kooistra and Helin, 2012). Currently, histone demethylases could be mainly categorized into two subfamilies based on homology and substrate specificity: LSD demethylases (LSD1-2) and Jumonji C (JmjC) domain-containing demethylases (JHDMs) (Markolovic et al., 2016). Dysfunction of histone demethylases has been observed in malignant diseases especially cancers such as colorectal cancer, bladder cancer and lung cancer (Hayami et al., 2011; Højfeldt et al., 2013). Harris et al. delineated the potential oncogenic role of LSD1 (KDM1A) in leukemia using the mouse model of MLL-AF9 leukemia (Harris et al., 2012). In another study, the authors showed that KDM2B was highly expressed in leukemia samples and played central role in the etiology and progression of acute myeloid leukemia (He et al., 2011). Thus, histone demethylases were considered as putative epi-targets for discovering anticancer agents. In the following section, we will discuss the successful applications of computational approaches in the field.

#### High throughput virtual screening

In order to pursue novel LSD1 inhibitors, Hazeldine et al. undertook the virtual screen strategy against Maybridge compound library. Sitemap was employed to assess the druggability of potential active chamber. Through high throughput virtual screen in GLIDE, the authors identified a total of 10 hits with GlideScore lower than −7.5 kcal/mol. The most effective compound (compound 42 in Figure 3) featuring amidoximes moiety displayed moderate *in vitro* activity with the IC_50_ value of 16.8 μM (Hazeldine et al., 2012). Later on, Sorna and co-workers reported structure-based docking studies with the ligand library containing 13 million compounds. High Throughput Virtual Screen (HTVS) protocol integrated in Schrödinger suite was applied and the database was subsequently refined by rule of five filters to weed out nonbinders and compounds with undesirable physicochemical parameters. Top 15% compounds were selected and re-ranked by combinatorial scoring with GLIDE, ICM, and GOLD to discard false positives. Based on chemical diversity analysis and visual inspection of initial docking results, 121 compounds were selected for biochemical validation and further medicinal chemistry optimization led to the identification of novel LSD1 inhibitor 12 (compound 43 in Figure 3) with the IC_50_ value of 0.013 μM (Sorna et al., 2013). Continued efforts have been made toward the discovery of potent, selective epi-probes against LSD1 and other histone demethylases (compounds 44–45,47 in Figure 3) based on computational approaches (Schmitt et al., 2013; Kutz et al., 2014; Roatsch et al., 2016). Chu et al. utilized GEMDOCK to screen the NCI database (~236,962 compounds) *in silico* and identified a selective KDM4A/KDM4B inhibitor (compound 48 in Figure 3) with the IC_50_ value at micromolar level (Chu et al., 2014). In 2016, Korczynska et al. performed molecular docking screens using ZINC fragment library (~600,000 commercially available fragments) in DOCK version 3.6 leading to the identification of 5-aminosalicylates as the KDM4C inhibitor with good ligand efficiency. Further docking analysis and fragment linking optimization yielded more potent inhibitor with K_i_ value of 43 nM (compound 49 in Figure 3) against KDM4C that highlighted the viable applications in fragment-based drug discovery (FBDD) (Korczynska et al., 2016).

#### 3D-QSAR pharmacophore modeling

In 2015, Zhou et al. presented pharmacophore-based ligand mapping strategy against LSD1 using refined SPECS database (~171,143 small molecules) in Discovery Studio version 2.5. 3D conformations of 37 compounds with known activities (22 compounds for training set and 15 compounds for test set) were generated and used to generate pharmacophore in HypoGen module. The reliability of the pharmacophore model was verified by Fischer randomization test and decoy set prediction. Through combinatorial pharmacophore mapping and optimized docking in database screening, the authors identified XZ-09 (compound 46 in Figure 3) as a selective LSD1 inhibitor with the IC_50_ value of 2.4 μM that may serve as a lead compound for further optimization (Zhou et al., 2015a).

## Reader

**Figure 4:**
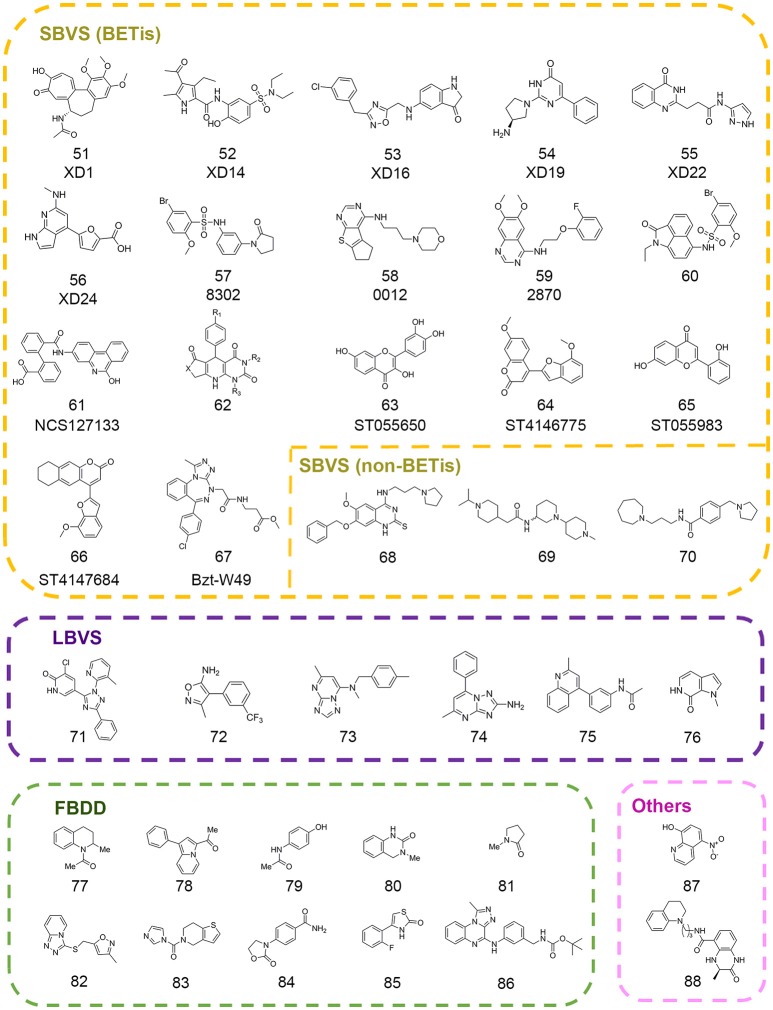
Chemical structures of epigenetic reader inhibitors mentioned in this review.

The posttranslational modifications on histone tails with different modification states are recognized by specific epigenetic readers, which recruit effector modules to stimulate different functions. Until now there are several well-characterized epigenetic readers including acetyl-lysine readers, methyl-lysine readers, methyl-arginine readers, and phospho-serine readers. Among them, lysine acetylation and methylation related readers were studied extensively as drug targets in epi-drug design and discovery. The acetyl-lysine readers consist of bromodomains and the tandem PHD domains (Lange et al., 2008; Filippakopoulos et al., 2012). And the readers associated with lysine methylation include PHD zinc finger domains, WD40, Tudor, double/tandem Tudor, MBT, Ankyrin Repeats, zf-CW, PWWP, and chromodomains (Kim et al., 2006; Collins et al., 2008; Musselman and Kutateladze, 2009; He et al., 2010; Rona et al., 2016; Schapira et al., 2017). Emerging evidence demonstrated the dysfunction of epigenetic readers is implicated in various diseases such as cancer, intellectual disability, aging, autoimmune disease, inflammation and acquired immune deficiency syndrome (Baker et al., 2008; Greer and Shi, 2012; Jung et al., 2015). So far, several successful compounds selectively targeting epigenetic reader domains have been reported and some of them enter into clinical studies (Greschik et al., 2017). Herein, we focus on the computer-aided drug discovery in epigenetic readers and review the successful examples to illuminate the advantages and potential applications of computational drug design and discovery in this field (Figure 4).

### Druggability prediction

Based on the complex crystal structure information of epigenetic readers with their relative substrates or small molecule inhibitors, the druggability of these targets could be easily predicted by computational methods. Many pragmatic programs have been developed and applied to explore potential drug-actionable pocket and assess the druggability of these binding sites (Halgren, 2009; Fauman et al., 2011). In 2011, Santiago et al. conducted the systematic druggability prediction for methyl-lysine binding proteins (Santiago et al., 2011). Based on the terms like steric volume, enclosure and hydrophobicity of the pocket, the Dscores of potential pockets were calculated using SiteMap. The results revealed that the druggability of different of methyl-lysine readers was highly variable dependent on backbone motion and intramolecular interactions, among which chromodomains, WDR domains and PWWP domains were more targetable than others like Tudor and PHD domains for small molecule inhibitors.

In 2012, to explore the druggability for bromodomains, the acetyl-lysine binders, Vidler et al. retrieved the available crystal structures of 33 human bromodomains from the Protein Data Bank (PDB) and evaluated druggability in SiteMap (Vidler et al., 2012). Among them, bromodomain, and extra-terminal (BET) family was predicted as the highly druggable target, which was already proved by small molecule inhibitors studies, but it could not represent the whole bromodomain families. The authors classified 49 bromodomains into eight categories based on common binding site features and found that only one of them showed the comparable druggability with the BET family including CECR2, FALZ (A/B), GCN5L2, PCAF, TAF1 (A/B)(2), and TAF1L(2). Other groups were predicted with low scores suggesting to be challenging for epi-drug discovery. Collectively, these work uncovered novel druggable readers that were less explored before, which provided new opportunities for drug discovery.

### Combinatorial *in silico* virtual screen approaches

With the rapid development of BET inhibitors, more complex crystal structures were obtained, which made structure-based virtual screen and chemical modifications more easily. Based on the well-known critical interactions between BET family and related inhibitors, many computational studies were performed to develop novel chemotypes for BET family.

In 2013, a high throughput virtual screening was performed with more than 7 million small molecules from the Dictionary of Natural Products, the ChEMBL database, and the ZINC database by Lucas and colleagues in order to discover novel inhibitors of BRD4(1) (Lucas et al., 2013). Based on standard precision and extra precision algorithm for molecular docking in GLIDE version 5.6, top-ranked 500 hits were clustered into 33 diverse categories. According to the prediction of several properties including physicochemical, pharmacokinetic, toxicological and binding promiscuity using various computational approaches, 22 candidate compounds were selected for further experimental validation. Finally, 7 compounds comprising 6 different novel scaffolds (compounds 51–56 in Figure 4) were identified with significant binding affinity. The subsequent resolved complex structures of BRD4(1) with XD14, XD1, and XD25 revealed the accurate binding modes consistent with the docking simulation.

In 2015, Allen et al. developed *in silico* screening approaches against kinases and bromodomains, which integrated machine learning and structure-based drug design strategies. At last several BRD4 inhibitors (compounds 57–58 in Figure 4) and one dual EGFR-BRD4 inhibitor (compound 59 in Figure 4) were identified (Allen et al., 2015). Similarly, Xue and co-authors performed another structure-based virtual screening against BET bromodomains (Xue et al., 2016). Approximately 10,000 compounds were firstly screened against BRD4(1) in GLIDE version 6.1. Through binding free energy assessment and cluster analysis, 15 representative compounds were chosen for biological evaluation. The results showed two compounds with benzo[*cd*]indol-2(1*H*)-one scaffold were identified as novel inhibitors targeting the BRD4(1). Before the optimization of this scaffold, binding modes of these two compounds were predicted by molecular docking in order to characterize the critical interactions. A 20 ns MD simulation was subsequently performed, which indicated the conformations were stable and reasonable for hit optimization. Further SAR analysis and resolved complex crystal structures provided guidance for hit optimization leading to the discovery of compound 85 (compound 60 in Figure 4) with high-potency biological activity.

Concomitantly, Tripathi et al. carried out a virtual screening against BRD2(2) using 1,700 compounds in NCI Diversity Set III library (Tripathi et al., 2016). The candidates were selected according to the free energy values, critical binding conformations, and ligand efficiency. Among them, crystal structure of compound NSC127133 (compound 61 in Figure 4) in complex with BRD2(2) was resolved, which displayed distinct structural features. In 2017, Ayoub et al. performed high throughput virtual screen with 6,000,000 compounds in ZINC database using the crystal structure of BRDT(1) (Ayoub et al., 2017). A dihydropyridopyrimidine scaffold (compound 62 in Figure 4) was identified with highly selectivity for BET family and submicromolar affinity for BRD4(1) and BRDT(1), which could be easily synthesized in one step.

With many new scaffolds uncovered from high throughput virtual screening, Raj et al. made an attempt to screen with flavonoids and derivatives instead of a common library with large collections of compounds (Raj et al., 2017). The followed ADMET properties analysis demonstrated the good drug-likeness properties of the identified compounds (compounds 63–66 in Figure 4) suggesting potential applications in the therapies for BET-related diseases. In another study, Deepak et al. designed three benzotriazepipne analogs using *in silico* tools with the aim to improve the selectivity between BET family members (Deepak et al., 2017). Combined with ensemble docking, MD simulation and binding energy calculation, compound Bzt-W49 (compound 67 in Figure 4) was synthesized and showed about 10-folds selectivity toward BRD4 compared to BRD2.

Besides the virtual screening efforts against BET family, drug discovery toward other readers has also progressed a lot in recent years. In 2016, a structure-based pharmacophore modeling combined with molecular docking were carried out to identify small molecule inhibitors of methyllysine reader protein Spindlin1 (Robaa et al., 2016). Several hits (compounds 68–70 in Figure 4) were subject to 2D-chemical similarity search and medicinal optimizations which improved the potency over 10-folds.

In addition to the *in silico* structure-based virtual screening against commercial libraries directly, the ligand-based computational methods would also help to improve the efficiency of virtual screening. In 2013, Vidler et al. carried out substructure searches for advanced enrichment of chemotypes in two branches (Vidler et al., 2013). For one thing, substructures that mimicked the acetyl-lysine moiety were searched in database. For another, similarity searching was performed to identify distinct chemotypes from known inhibitors using pharmacophore models, shape-based 2D fingerprint searches. The extensive set of substructures obtained was submitted to molecular docking in eMolecules database and manual selection for further experimental validation. Finally six novel hits (compounds 71–76 in Figure 4) including four unprecedented acetyl-lysine mimetics were identified. Structure-guided chemical modifications were performed based on complex crystal structures to improve the potency. In 2016, Hugle et al. screened PurchasableBoX library to select analog of previously identified bromodomain inhibitor XD14 (compound 52 in Figure 4) (Hügle et al., 2016). Several candidates were used to explore the SAR of XD14 and additional structural features of BRD4 through DFT calculation, atom-based QSAR and ligand-based pharmacophore, which offered the guidance for the development of novel BRD4(1) inhibitors.

### Fragment-based drug discovery

Fragment-based drug discovery has been widely practiced in drug discovery and some FBDD-derived drugs have entered into the clinical study (Erlanson et al., 2016). Many CADD integrated tools have been designed for scaffold replacement and fragment growing such as Molecular Operating Environment (MOE) developed by Chemical Computing Group, which could accelerate the pace of FBDD-guided drug discovery. In 2012, Chung et al. firstly built a fragment library that contained substructures with acetyl-lysine mimetic functional groups to identify novel BET inhibitors (Chung et al., 2012). The library was filtered to eliminate unsuitable substructures based on “rule of three” and predicted p*K*_a_ values. The remaining fragments were clustered and then representative members were selected in each cluster according to docking results. Coupled with follow-up experiments, Chung and colleagues identified several compounds (compounds 77–81 in Figure 4) with two novel fragment scaffolds, which significantly extended the chemotypes of current inhibitors.

In 2013, Zhao et al. built a fragment library to discover novel BRD4 inhibitors (Zhao et al., 2013a). The fragment compounds in ZINC database were filtered by particular rules including molecular weight ≤ 250 Da, rotatable bonds ≤ 5, log *P* ≤ 3.5, and 1 ≤ smallest set of small ring ≤ 4. According to the Tanimoto similarity calculated in Pipeline Pilot, 487 representative fragments were purchased to build the fragment library. Through molecular docking with established in-house library and crystallization experiments, 9 fragments were identified in the binding pocket of BRD4(1) in the solved crystal structures and four of them (compounds 82–85 in Figure 4) were presented in Figure 4. Further pharmacokinetic study showed the great potential for further drug development. In 2017, Ali et al. performed docking-based virtual screening with fragment-like database containing nearly 800,000 compounds from ZINC database in an effort to pursue BRD4 inhibitors (Ali et al., 2017). Finally, the authors unveiled the discovery of a novel scaffold (compound 86 in Figure 4) contained [1,2,4]triazolo[4,3-α]quinoxaline as BET inhibitors. Several rounds of chemical modification led to the synthesis of analogwith high potency and improved pharmacokinetic properties.

### Target-specific scoring function

Considering the better druggability for BET family, many efforts were devoted to the discovery of novel BET inhibitors. However, the performance of either virtual screening or high throughput screening varies and shows high rate of false positives, which restricts the applications in this field. In order to improve enrichment factor in screening, a BRD4-specific score named BRD4LGR was developed through machine-learning-assisted approach by Xing et al. (Xing et al., 2017). First-round virtual screening was performed in GLIDE version 5.6 and 453 compounds were selected for *in vitro* evaluation resulting in a high false positive rate of 95%. Based on the first-round screening results and other reported studies, structure and activity data of 814 compounds was collected to construct specific scoring function. The authors identified critical molecular interaction features from reported complex structures and established logistic regression model to correlate the interaction features to potencies. Compared with GLIDE and PMF, BRD4LGR discriminated BRD4 inhibitors and non-inhibitors more effectively with high specificity and sensitivity. A second-round virtual screening using BRD4LGR identified 15 new active compounds with a lower FP rate at 85%. Beyond this, BRD4LGR was capable of interpreting key structure-activity relationships of BRD4 inhibitors, which would be quite valuable for chemistry optimization.

In a follow-up study, Jiang et al. employed virtual screening strategy with an in-house compound library containing 887 FDA-approved drugs using BRD4LGR scoring model (Jiang et al., 2017). The docking-based virtual screening coupled with similarity-based analog searching led to the discovery of nitroxoline (compound 87 in Figure 4) as a potent and novel BET inhibitor that was previously used to treat urinary tract infections. The successful application of BRD4LGR suggested potential use of nitroxoline in the treatment of BET family-related diseases.

### Quantum mechanical calculations

Quantum mechanical calculations are commonly used to understand the nonbonding interactions, such as cation-π and hydrogen bond interactions. In order to explain the different affinity of 1,5-naphthyridine derivatives, Mirguet et al. carried out in vacuo QM calculations to calculate the bound conformations of several derivatives in their complex with BRD2 (Mirguet et al., 2014). The results showed that the differences in internal geometric energy might account for differences in relative bioactivity.

Besides, quantum mechanical calculations could be applied in combination with other computational studies in epi-probes discovery. In 2014, Rooney et al. identified two CREBBP bromodomain inhibitors with weak activity by *in silico* screen and biochemical assays (Rooney et al., 2014). Further structure-based chemical modifications led to the compound *(R)*-1 (compound 88 in Figure 4) with the IC_50_ value of 758 nM. The complex structure of (*R*)-1 and CREBBP bromodomain revealed an induced-fit pocket that didn't exist in apo-form. *(R)*-1 formed a cation-π interaction with R1173 to maintain the stability of the conformation. In an effort to rationalize the importance of the cation-π interaction, the authors undertook MD simulation in which the cation-π interaction was observed for 40% of the trajectory time. Then the strength of cation-π interaction was estimated by DFT calculations with the strength value of 3.2–4.7 kcal mol^−1^ in accordance with the experimentally measured average strengths involving lysine or arginine. Meanwhile, DFT calculations were also applied to confirm the significance of internal hydrogen bound in ligand conformation which were also applicable in other studies.

## Protein-protein interaction

**Figure 5:**
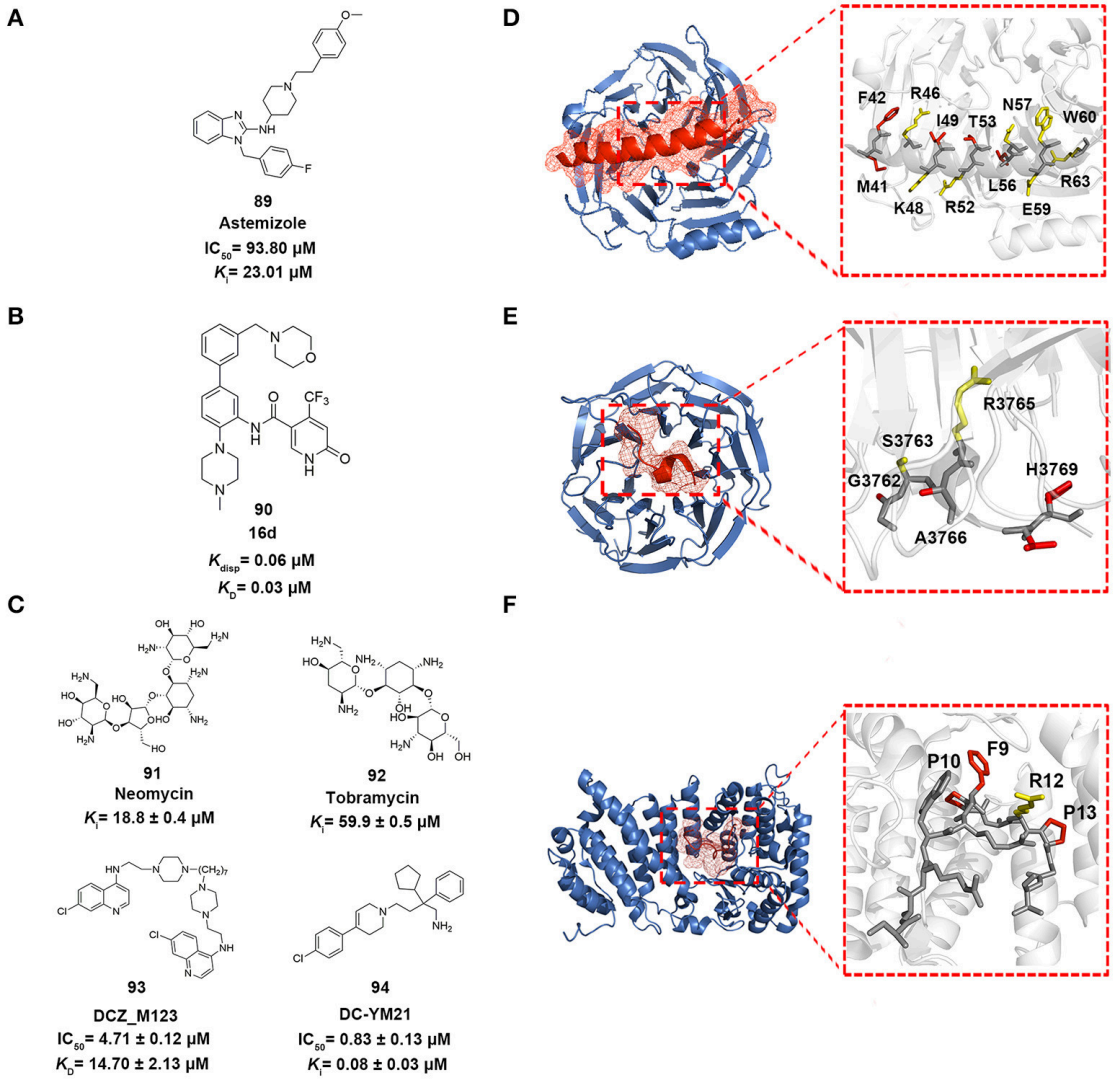
Epigenetic PPI inhibitors. **(A–C)** Chemical structures of the inhibitors mentioned in this review. **(D–F)** The detailed interactions patterns of EZH2-EED (PDB code: 2QXV), WDR5-MLL1 (PDB code: 3EG6), and Menin-MLL1 (PDB code: 4GQ6). EED, WDR5, and Menin are represented in surface contours. The side chains of key residues involved in hydrophobic interactions are depicted in red while the ones involved in polar interactions are depicted in yellow.

Epigenetic enzymes from the same protein subfamily often share similar catalytic core pockets and cofactors within family members, thus making it quite difficult to discover and design a selective inhibitor. A growing body of evidence suggests that a variety of protein–protein interactions (PPIs) are indispensable for integrity and oncogenic function of epigenetic enzymes. Therefore, these PPIs appear to be alternative drug targets to modulate chromatin state in epigenetic drug discovery. Due to the unique structural features of PPIs, which have large and flat contact surface and the lack of well-defined pockets, it remains challenging to explore small molecule inhibitors targeting epigenetic interactome (Wells and McClendon, 2007). However, with high-resolution protein complex structures resolved, advanced computational tools developed and renewed understanding of PPIs mechanisms, great progress has been made in the development of small molecule inhibitors (Scott et al., 2016). Here, we focus on the application of CADD methods, including structure-based virtual screening, scaffold hopping, structure-based pharmacophore modeling, and ligand-based pharmacophore profiling in the discovery and design of small molecule inhibitors targeting important epigenetic PPIs including EZH2-EED, WDR5-MLL1, and Menin–MLL1 (Figure 5).

## EZH2-EED

Polycomb repressive complex 2 (PRC2) specifically trimethylates lysine 27 at histone H3, which is one of the cardinal marks for transcriptional repression (Simon and Kingston, 2009). Enhancer of zeste homolog 2 (EZH2) is the catalytic subunit of PRC2, which requires two additional subunits embryonic ectoderm development (EED) and suppressor of zeste 12 (SUZ12) for full functional activity (Czermin, 2002; Cao and Zhang, 2004). Aberrant PRC2 activity has been reported in the initiation and progression of wide range of cancers (Chang and Hung, 2012). Thus drug design and discovery targeting the PRC2 complex formation represents the unique strategy in chemical intervention.

Drug repositioning is an increasingly attractive strategy widely applied in biopharmaceutical companies to identify alterative therapeutic indications from approved drugs (Ashburn and Thor, 2004). In 2014, in order to pursue EZH2-EED inhibitors, Kong et al. utilized structure-based virtual screening approach to enrich the hits from in-house compound library containing *ca*. 1,000 existing drugs (Kong et al., 2014). The standard precision and extra precision mode in GLIDE version 5.5 were subsequently employed to perform docking-based virtual screening leading to the identification of astemizole (compound 89 in Figure 5), a FDA-approved antihistamine drug as moderate EZH2-EED inhibitor with *K*_i_ value of 23.0 μM. Further biophysical assays and cellular studies demonstrated the competitive MOA of astemizole and its inhibition for intracellular PRC2 activity.

## WDR5-MLL1

Mixed lineage leukemia 1 (MLL1) is the histone methyltransferase responsible for the H3K4 methylation. MLL1 interacts with many chaperons including WD repeat-containing protein 5 (WDR5), a common unit that is essential for the integrity of the catalytic core complex (Dou et al., 2006). Therapeutically targeting WDR5-MLL1 interaction by peptidomimetic inhibitors has been demonstrated as a promising strategy for MLL fusion-mediated acute leukemogenesis (Karatas et al., 2013).

In 2016, Getlik and co-workers designed focused library *in silico* guided by crystal structure information and initial SAR exploration on previously identified benzamides scaffold (Getlik et al., 2016). An exhaustive virtual enumeration was performed in Pipeline Pilot to search all accessible building blocks containing benzamides moieties. The set of compounds with poor physicochemical properties were removed by OICR HTS filters. About 1,200 acyl halides and 9,000 acids/esters were enumerated and used for further medium-throughput virtual screening. Subsequently, molecular docking was performed in GLIDE with one H-bond constraint to the side chain of S91 in WDR5. Through overall consideration of the docking score, binding pose, structural complexity and synthetic difficulty, 50 representative compounds were selected by visual inspection and prioritized as candidates for synthesis and verification. Finally, 4-(trifluoromethyl)pyridin-2(1*H*)-one moiety was discovered as better alternative in replacement of the benzamide moiety. Among the derivatives, the optimized antagonist 16 days (compound 90 in Figure 5) was the most potent inhibitor against WDR5-MLL1 with the *K*_disp_ value of 60 nM, which offered novel therapeutic options in the treatment of leukemia harboring MLL fusion proteins.

### Menin-MLL1

The oncoprotein MLL1 can directly associate with cofactor Menin through N-terminal 43 amino acids including two Menin-binding motifs (MBMs), MBM1 (*K*_d_ = 53 ± 4.2 nM) and MBM2 (*K*_d_ = 1.4 ± 0.42 μM) (Grembecka et al., 2010). Menin-MLL1 interaction is required for oncogenic function of MLL fusion proteins and contributes to related leukemia pathogenesis (Yokoyama and Cleary, 2008; Huang et al., 2012). Thus, the Menin-MLL1 PPI interface has been spotlighted as a potential target for epi-drugs development against MLL-mediated leukemia.

In 2014, Li et al. employed structure-based pharmacophore modeling targeting the Menin–MLL1 interface based on the interaction patterns of Menin and MBM1 complex structure (PDB ID: 4GQ6) (Shi et al., 2012; Li et al., 2014). 10 best pharmacophore models were generated in Discovery Studio 3.0, considering the features of HBD, HBA, and hydrophobic group. Based on overall consideration of the fitness score in generated models, excluded volumes and hot spots analysis, one pharmacophore model with two hydrophobic groups and a hydrogen bond acceptor was selected as a query for follow-up virtual screening. Then an in-house library comprising 900 exiting drugs was built and queried by the constructed pharmacophore model. 29 compounds were finally selected for biochemical verification. Among them, two aminoglycoside antibiotics, neomycin and tobramycin (compounds 91–92 in Figure 5), were identified as Menin–MLL1 inhibitors in fluorescence polarization competition assay with binding affinities of 18.8 and 59.9 μM, respectively. Thermal shift assay and isothermal titration calorimetry validated the direct interactions between the two antibiotics and Menin. Molecular docking analysis indicated these antibiotics competitively occupied the binding site of MLL1 in the central cavity of Menin.

In 2016, Xu and co-workers conducted the structure-based molecular docking and ligand-based pharmacophore modeling to obtain Menin-MLL1 inhibitors (Xu et al., 2016). To establish the ligand data set, 74 previously reported inhibitors classified into three categories were collected and 5,000 decoy compounds were generated based on 10 compounds with best potency by DecoyFinder (Cereto-Massagué et al., 2012). For one thing, molecular docking with various constrained conditions was subsequently performed in GLIDE. According to the Glide score and enrichment factor (EF) values, non-constraint SP docking approach performed best and was more appropriate for SBVS that could well distinguish known inhibitors from decoys for Menin-MLL1 inhibitors. For another, ligand-based pharmacophore models with 4–6 pharmacophore features (HBA, HBD, hydrophobic group, aromatic ring and positively or negatively charged group) were generated from those collected inhibitors with pIC_50_ > 5.0. 3D-QSAR models were then developed based on the built pharmacophore models through partial least-squares (PLS) regression analysis. Through the joint LBVS and SBVS computational strategies, five compounds with novel scaffolds were identified as Menin-MLL1 inhibitors validated by fluorescence polarization assay. Among them, DCZ_M123 (compound 93 in Figure 5) showed the most potent inhibitory activity *in vitro* with the IC_50_ value of 4.7 μM and could effectively inhibit the growth of MLL leukemia cells by impairing the Menin-MLL1 interaction in cell-based assays.

Scaffold hopping was proposed as a promising strategy to look for novel molecular entities with similar three dimensional conformations and properties (Schneider et al., 1999). As a shape-based three dimensional structure superposition method, it has been extensively used to generate potential alternatives of known compounds based on the bioisosteric replacement of core motif within molecules (Sun et al., 2012; Lamberth, 2017). In 2016, Yue et al. applied a shape-based scaffold hopping approach to reposition approved drugs targeting the Menin-MLL1 interaction (Yue et al., 2016). In the study, reported bioactive conformations of representative Menin-MLL1 inhibitors MI-2-2 and MIV-6R (PDB code 4GQ4 and 4GO8, respectively) were used as query (Shi et al., 2012; He et al., 2014). An in-house library comprising ~1,600 existing drugs was aligned onto the query to perform 3D similarity searching using SHAFTS (Liu et al., 2011; Lu et al., 2011). A set of 12 top ranked compounds with SHAFTS similarity scores >1.2 (maximum 2.0) were selected for primary validation, which indicated that loperamide, previously used as anti-diarrhea agents, showed weak inhibition with the IC_50_ value of 69 μM. Further molecular docking analysis and medicinal chemistry optimization led to the identification of more potent loperamide-derived analog. Among them, DC_YM21 (compound 94 in Figure 5) presented nanomolar inhibitory activity of the same order of magnitude as the reported inhibitor MI-2-2.

## Future perspectives

Computational methods are indispensable and creditable tools in both academia and industry that undoubtedly streamline the epi-drug and epi-probe discovery process. The focal point of this review is the state of art of CADD methods in epi-drug design and discovery framework over the past decades. Tremendous progress has been achieved in epigenetic drug discovery based on *in silico* approaches as we have mentioned above which unequivocally draws a positive picture in the field. However, it is widely accepted that these aforementioned hit-finding methodologies are far from perfect and not omnipotent in all situations. There are still formidable challenges that need to be overcome which limit the effective applications of current computational methods. Firstly, current molecular docking scoring functions rank the compounds collections with inherent poor prediction accuracy in novel target drug discovery whose function has just been unraveled not long ago (Sable and Jois, 2015). Secondly, traditional docking algorithms fail to take complicated factors into full consideration like protein flexibility, solvation, entropy, and dynamic inclusion of water molecules (Clark, 2008; Lavecchia and Di Giovanni, 2013). Thus, it's difficult to precisely predict the absolute binding energy for ligand-protein interactions based on current methodologies. There are some reviews that investigate protein flexibility in detail (Barril and Fradera, 2006). However, the current computational methodology considering this issue is time-consuming that needs to be further improved. Thirdly, despite the fact that epigenetic enzymes have been actively pursued as potential drug targets, there is still conspicuous lack of potent chemical probes for a large number of knotty targets like HATs and epigenetic protein-protein interactions, which needs to be further explored. For these less well-studied epi-targets, there are few inhibitors with limited diversity of scaffolds ever reported that hinders the ligand-based drug design and development. For instance, PRMT5-MEP50 complex formation could enhance the stability and activity of PRMT5 and the PPI is essential for cancer cell invasion in lung cancer and breast cancer (Chen et al., 2017). Heterooctameric PRMT5-MEP50 complex structure has been resolved which enables structure-based drug design. Nonetheless, no chemical probes have ever been reported for such novel targets. Fourthly, the bioactivities of identified inhibitors vary considerably due to different assay platforms in differ different labs. Some of the reported inhibitors belong to pan-assay interference compounds and present non-specific interactions that have not been carefully examined (Dahlin et al., 2017). Overinterpretation of these results leads to misleading readouts and would go to the cul-de-sac in drug discovery process. Taken together, there are still many problems left unsolved which encourage the researcher to devote more drug discovery efforts in order to fill the vacancy in this field. To tackle with these issues, integrated SBVS and LBVS approaches should be applied to counterbalance their own limitations in a parallel manner in virtual screen campaigns. As for novel targets with fewer inhibitors ever reported, computational methods should be applied in synergy with experimental approaches. Multidisciplinary efforts shall be devoted to generate more diverse machine learning datasets for the establishment of target-customized scoring functions, which in turn help to exploit chemical space available in database as thoroughly as possible. Meanwhile, the researchers should carefully examine the biological data before interpreting the biological results. This appeals to the researchers to develop a reliable experimental platform to standardize current biochemical assays. It could be expected that with rapid development of computational power and methodologies, more epi-drugs and epi-probes will be developed in the near future, which could not only help to uncover the elusive role of each node in epigenetic regulatory network but also guide optimum therapeutic options in the treatment of epigenetic-related diseases.

## Author contributions

WL, RZ, HJ, HZ, and CL wrote the manuscript. WL, RZ, and CL organized and revised the manuscript. All authors were involved in the preparation of the manuscript and approved the final version.

### Conflict of interest statement

The authors declare that the research was conducted in the absence of any commercial or financial relationships that could be construed as a potential conflict of interest.
